# Advanced glycation end products-induced insulin resistance involves repression of skeletal muscle GLUT4 expression

**DOI:** 10.1038/s41598-018-26482-6

**Published:** 2018-05-25

**Authors:** Danilo C. Pinto-Junior, Karolline S. Silva, Maria L. Michalani, Caio Y. Yonamine, João V. Esteves, Nelly T. Fabre, Karina Thieme, Sérgio Catanozi, Maristela M. Okamoto, Patricia M. Seraphim, Maria L. Corrêa-Giannella, Marisa Passarelli, Ubiratan F. Machado

**Affiliations:** 1 Departament of Physiology and Biophysics, Institute of Biomedical Sciences, Universidade de São Paulo, São Paulo, Brazil; 2 Laboratório de Carboidrato e Radioimunoensaio (LIM-18), Hospital das Clinicas HCFMUSP, Faculdade de Medicina, Universidade de São Paulo, São Paulo, Brazil; 3 Laboratório de Lípides (LIM-10), Hospital das Clinicas HCFMUSP, Faculdade de Medicina, Universidade de São Paulo, São Paulo, SP Brazil; 4 Department of Physiotherapy, Faculty of Sciences and Technology, Universidade Estadual Paulista, Presidente Prudente, São Paulo, Brazil; 5 Programa de Pós-Graduação em Medicina, Universidade Nove de Julho (UNINOVE), São Paulo, Brazil

## Abstract

Little is known about advanced glycation end products (AGEs) participation in glucose homeostasis, a process in which skeletal muscle glucose transporter GLUT4 (*Scl2a4* gene) plays a key role. This study investigated (1) the *in vivo* and *in vitro* effects of AGEs on *Slc2a4*/GLUT4 expression in skeletal muscle of healthy rats, and (2) the potential involvement of endoplasmic reticulum and inflammatory stress in the observed regulations. For *in viv*o analysis, rats were treated with advanced glycated rat albumin (AGE-albumin) for 12 weeks; for *in vitro* analysis, soleus muscles from normal rats were incubated with bovine AGE-albumin for 2.5 to 7.5 hours. *In vivo*, AGE-albumin induced whole-body insulin resistance; decreased (~30%) *Slc2a4* mRNA and GLUT4 protein content; and increased (~30%) the nuclear content of nuclear factor NF-kappa-B p50 subunit (NFKB1), and cellular content of 78 kDa glucose-regulated protein (GRP78). *In vitro*, incubation with AGE-albumin decreased (~50%) the *Slc2a4*/GLUT4 content; and increased cellular content of GRP78/94, phosphorylated-IKK-alpha/beta, nuclear content of NFKB1 and RELA, and the nuclear protein binding into *Slc2a4* promoter NFKB-binding site. The data reveal that AGEs impair glucose homeostasis in non-diabetic states of increased AGEs concentration; an effect that involves activation of endoplasmic reticulum- and inflammatory-stress and repression of *Slc2a4*/GLUT4 expression.

## Introduction

Advanced glycation end products (AGEs) have been extensively implicated in the genesis and progression of diabetes-related complications (Brownlee 2001). Additionally, AGEs might also contribute to glycemic impairment, by activating oxidative, endoplasmic reticulum (ER) and inflammatory stress in tissues related to insulin-regulated plasma glucose disposal^1^. Regarding that, some studies have suggested that AGEs can impair adipocyte glucose disposal *in vitro*^2,3^. However, in the major site of insulin-regulated glucose disposal, the skeletal muscle^4^, the effects of AGEs upon the glucose uptake markers, both *in vivo* and *in vitro*, are not known.

In muscles, insulin-stimulated glucose uptake is performed through the solute carrier family 2, facilitated glucose transporter member 4 (GLUT4), which is rapidly translocated to the plasma membrane in response to the hormone^5^. Besides, muscle contraction can also stimulate GLUT4 translocation, which operates in addition to insulin^6^. Although reduced GLUT4 translocation impairs skeletal muscle glucose uptake characterizing insulin resistance^7^, long-term established insulin resistance has currently been related to a defective glucose transporter gene and/or protein expression^8,9^.

AGEs interact with AGER (advanced glycosylation end product-specific receptor, former RAGE), activating the NFKB (nuclear factor NF-kappa-B) pathway and the expression of inflammatory genes^1,10^. By inducing inflammation, AGEs trigger ER-stress, which once activated, elicits the unfolded protein response (UPR) in order to restore cellular homeostasis^1,10^. In this process, a rapid activation of several chaperone networks is an early event, which can be monitored by the expression of GRP78 (78 kDa glucose-regulated protein) and GRP94 (endoplasmin/94 kDA glucose-regulated protein) proteins^11,12^. AGE/AGER interaction, directly or indirectly (via UPR), activates the inflammatory pathway, modulating distinct steps such as phosphorylation of IKKA and IKKB (inhibitor of nuclear factor kappa-B kinase, subunits alpha and beta, respectively), degradation of IKBA and IKBB (nuclear factor kappa-B inhibitor alpha and beta, respectively), and nuclear translocation of NFKB^11,12^. Recently, it was definitely demonstrated that, in nucleus, both NFKB1 (nuclear factor NF-kappa-B p105 subunit) and RELA (nuclear factor NF-kappa-B p65 subunit) bind into the promoter region of *Slc2a4* gene, repressing its transcription, and hence decreasing GLUT4 expression^13^. Thus, in a converging way, AGEs-stimulated ER and inflammatory stress might reduce skeletal muscle glucose disposal, contributing to glucose homeostasis impairment.

On the other hand, the interaction of AGEs with DDOST (dolichyl-diphosphooligosaccharide-protein glycosyltransferase 48 kDa subunit, former AGER1) can counterbalance the AGER-mediated deleterious pathways^14^ by inducing antioxidant mechanisms.

In diabetes (DM), AGE formation increases as a consequence of hyperglycemia and oxidative stress; however, their own effects on glucose homeostasis have not yet been investigated. Besides, AGEs can also be obtained from exogenous sources, such as high fat/sugar heat-processed foods, abundant in modern diets^15^, reinforcing the importance of knowing its effects on glucose homeostasis. We hypothesized that AGEs, independent of the occurrence of hyperglycemia, can modulate skeletal muscle glucose disposal by altering GLUT4 expression, thus impairing glucose homeostasis. Considering that, the present study aimed to investigate: (1) *in vivo*, the chronic effects of AGEs on glucose homeostasis, and on the expression of *Slc2a4*/GLUT4 and molecular markers of both UPR and inflammation in soleus muscle; and (2) *in vitro*, the direct effects of AGEs on expression of *Slc2a4*/GLUT4 and molecular markers of both UPR and inflammation in soleus muscle.

## Results

### Treatment of rats with AGE-albumin induced insulin resistance

**Figure 1:**
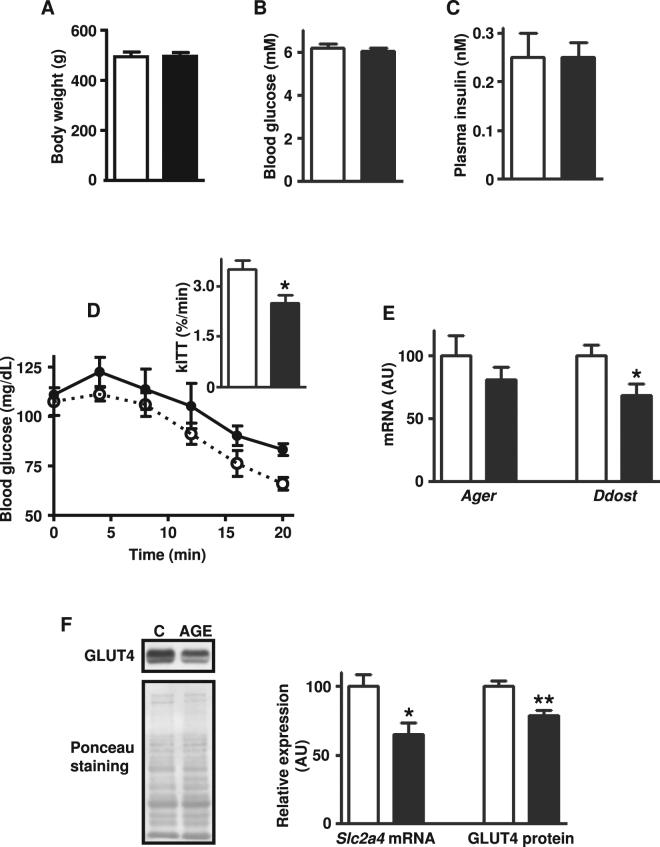
Treatment of rats with advanced glycated albumin induces insulin resistance and represses *Slc2a4*/GLUT4 expression. Body weight gain (**A**), blood glucose (**B**), plasma insulin (**C**), blood glucose decay and constant rate (kITT) during insulin tolerance test (**D**), skeletal muscle expression of advanced glycosylation end product-specific receptor (*Ager*), and of dolichyl-diphosphooligosaccharide-protein glycosyltransferase non-catalytic subunit (*Ddost*) mRNAs (**E**), solute carrier family 2 member 4 (*Slc2a4*) mRNA, and solute carrier family 2 facilitated glucose transporter member 4 (GLUT4) protein (**F**) were measured in rats chronically treated with advanced glycated- (AGE; black bars; closed circles) or control- (C; white bars; open circles) rat albumin. Representative autoradiograms and Ponceau S staining of respective lanes, used as protein loading control, are shown in (**F**). Data are mean ± SEM of 4 (panel D) or 7 (panels A–C,E and F) animals. Means were compared by unpaired two-tailed *t* test. *P < 0.05 and **P < 0.01 vs control-albumin.

As previously described^16^, twelve-week treatment with AGE-albumin reduced the glucose decay constant rate (kITT) during the insulin tolerance test (Fig. 1D); however, the treatment did not alter rat body mass gain, blood glucose or plasma insulin concentrations (Fig. 1A–C). Our hypothesis that this insulin resistance involves a direct effect of AGE-albumin in soleus muscle was supported by the detection of reduced expression of *Ddost* gene (Fig. 1E). Besides, participation of reduced skeletal muscle glucose disposal in the whole-body insulin resistance was evinced by the repression of *Slc2a4* mRNA and GLUT4 protein expression (Fig. 1F).

### Treatment of rats with AGE-albumin induced markers of reticulum endoplasmic and inflammatory stress in skeletal muscle

**Figure 2:**
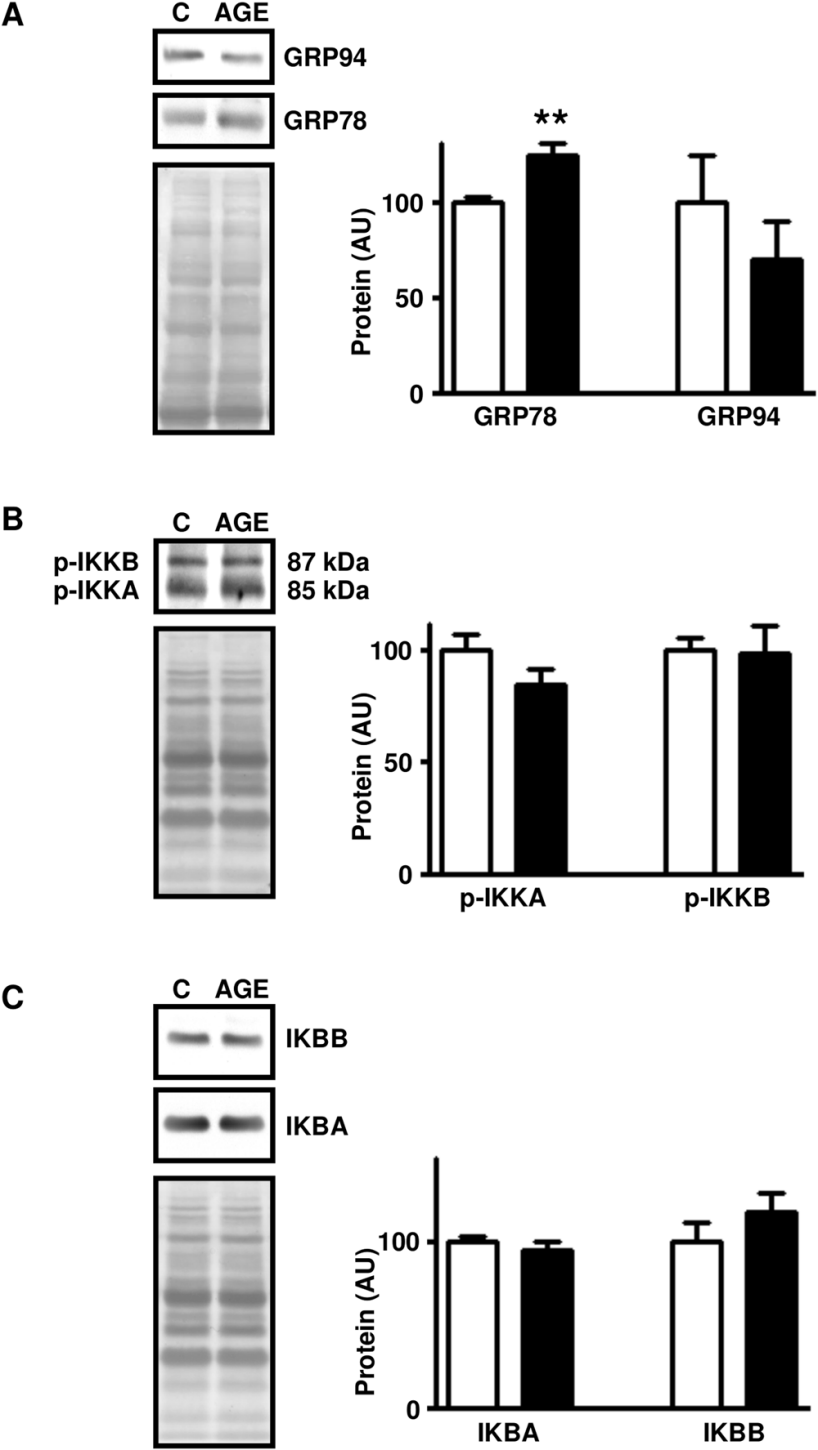
Treatment of rats with advanced glycated albumin activates markers of endoplasmic reticulum and inflammatory stress in skeletal muscle. Markers of endoplasmic reticulum stress: 78 kDa glucose-regulated protein (GRP78) and 94 kDa glucose-regulated protein (GRP94) (**A**), and markers of inflammatory stress: inhibitor of nuclear factor kappa-B kinase subunits alpha (IKKA) and beta (IKKB) (**B**), and nuclear factor kappa-B inhibitors alpha (IKBA) and beta (IKBB) (**C**) were measured in skeletal muscles of rats chronically treated with advanced glycated- (AGE; black bars) or control- (C; white bars) rat albumin. In each panel, representative autoradiograms and Ponceau S staining of respective lanes, used as protein loading control, are shown. Data are mean ± SEM of 7 animals. Means were compared by unpaired two-tailed *t* test. **P < 0.01 vs control-albumin.

**Figure 3:**
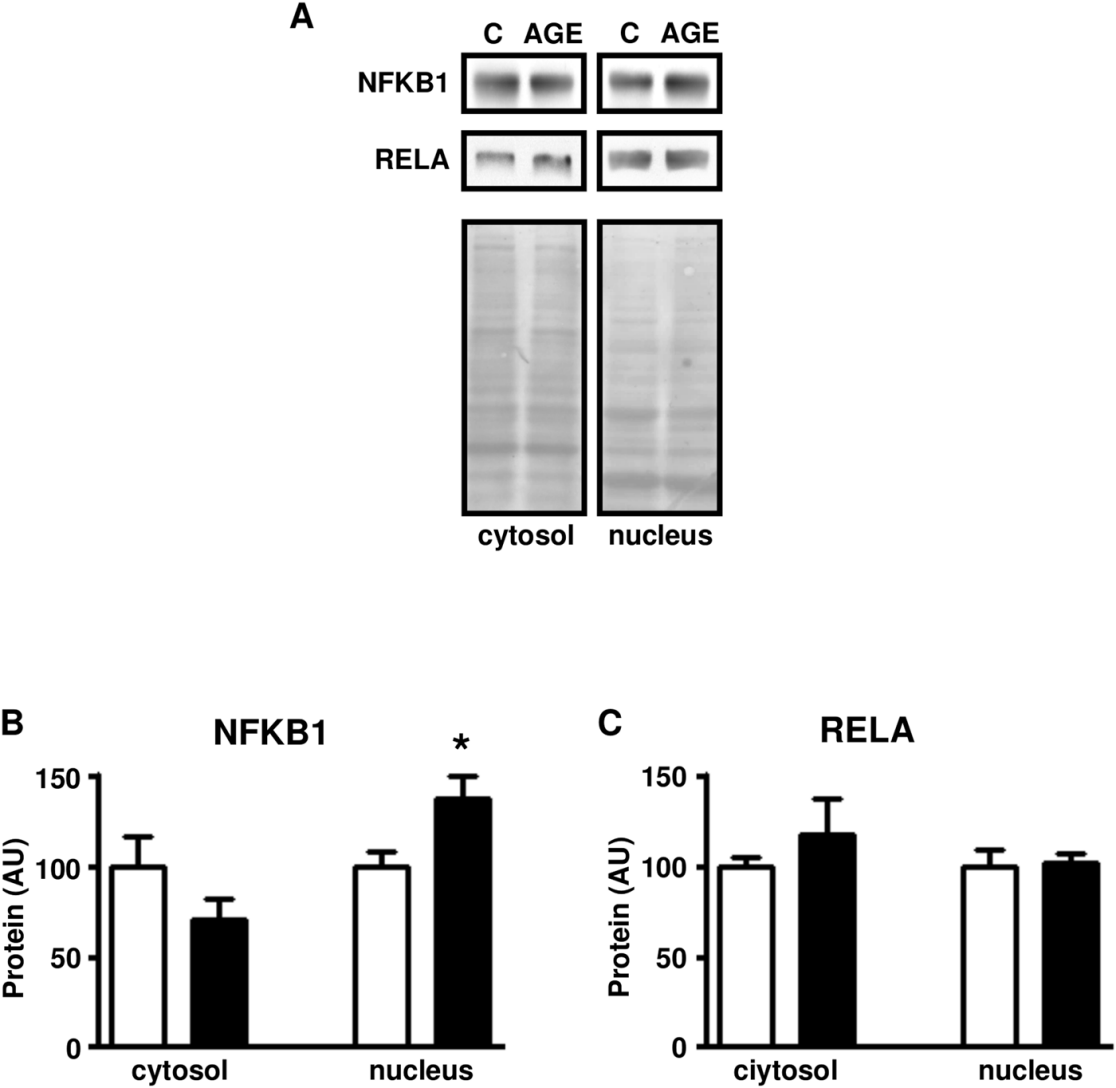
Treatment of rats with advanced glycated albumin activates nuclear factor NF-kappa-B in skeletal muscle. Nuclear factor NF-kappa-B p50 protein (NFKB1) (**A**,**B**) and nuclear factor NF-kappa-B p65 protein (RELA) (**A**,**C**) contents were measured in cytosolic and nuclear subcellular fractions from skeletal muscles of rats chronically treated with advanced glycated albumin- (AGE; black bars) or control- (C; white bars) rat albumin. In (**A**), representative autoradiograms and Ponceau S staining of respective lanes, used as protein loading control, are shown. Data are mean ± SEM of 7 animals. Means were compared by unpaired two-tailed *t* test. *P < 0.05 vs control-albumin.

*In vivo* treatment with AGE-albumin increased the expression of GRP78 chaperone (Fig. 2A), revealing ER stress activation. Phosphorylation of IKKA and IKKB (Fig. 2B) and IKBA and IKBB content (Fig. 2C) were unchanged; however nuclear content of NFKB1 (Fig. 3) was increased, indicating the activation of a fundamental inflammatory pathway.

### *In vitro* treatment of rat skeletal muscle with AGE-albumin repressed GLUT4 expression

**Figure 4:**
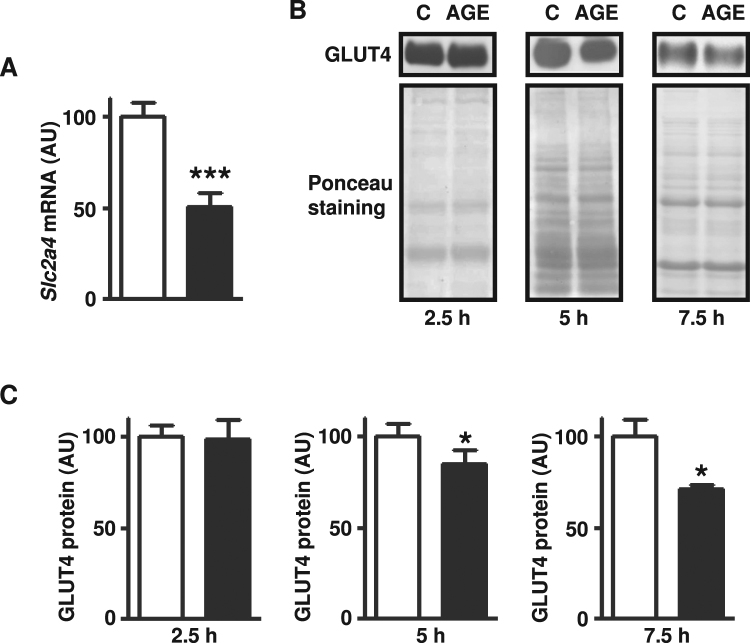
*In vitro* treatment of rat skeletal muscle with advanced glycated albumin represses *Slc2a4*/GLUT4 expression. Solute carrier family 2 member 4 (*Slc2a4*) mRNA (**A**) and solute carrier family 2 facilitated glucose transporter member 4 (GLUT4) protein (**B**,**C**) were measured in skeletal muscles of untreated rats incubated with advanced glycated- (left muscle; AGE; black bars) or control- (right muscle; C; white bars) bovine albumin during 2.5 hours. For GLUT4 analysis, incubation periods were additionally extended to 5 and 7.5 hours, and experiments of each period were performed separately (**B**,**C**). In (**B**), representative autoradiograms and Ponceau S staining of respective lanes, used as protein loading control, are shown. Data are mean ± SEM of 5 (GLUT4 protein) or 8 (*Slc2a4* mRNA) animals. Means were compared by paired two-tailed *t* test. *P < 0.05 and ***P < 0.001 vs control-albumin.

To confirm a direct effect of AGE-albumin in skeletal muscle, soleus muscles from untreated healthy rats were incubated with AGE- or C-albumin. After 2.5-hour incubation, AGE-albumin decreased (50%) the *Slc2a4* mRNA expression (Fig. 4A); but this period was not enough to decrease the total amount of cellular GLUT4 protein content. Nevertheless, extension of the incubation period to 5 and 7.5 hours (Fig. 4B and C) induced a progressive decrease in GLUT4 expression, which reached a 25% reduction (P < 0.05) after 7.5-hour challenge with AGE-albumin.

Considering that AGE-albumin treatment might also impair GLUT4 vesicles translocation, we tested the glucose uptake in muscles incubated for 2.5 hours, when GLUT4 content was still unaltered. The results revealed that neither the basal (2.017 ± 0.185 *vs* 2.15 ± 0.21 μmol/g tissue, control- *vs* AGE-albumin, respectively; n = 6; P = 0.979) nor the insulin-stimulated (4.80 ± 0.326 *vs* 4.62 ± 0.213 μmol/g tissue, control- *vs* AGE-albumin, respectively; n = 6; P = 0.948) glucose uptake was altered by the presence of AGE-albumin, although the positive effect of insulin (P < 0.001) was observed in both conditions.

### *In vitro* treatment of rat skeletal muscle with AGE-albumin induced endoplasmic reticulum and inflammatory stress markers

**Figure 5:**
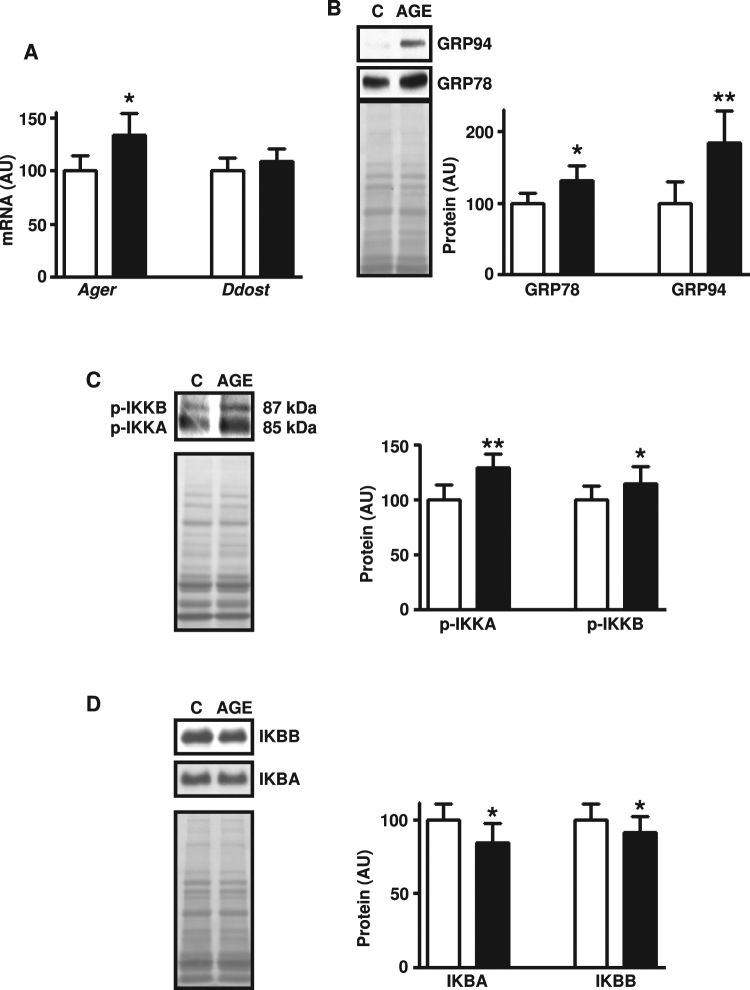
*In vitro* treatment of rat skeletal muscle with advanced glycated albumin activates endoplasmic reticulum stress and inflammatory pathways. Advanced glycosylation end product-specific receptor (*Ager*) and dolichyl-diphosphooligosaccharide-protein glycosyltransferase non catalytic subunit (*Ddost*) mRNAs (**A**); 78 kDa glucose-regulated protein (GRP78) and 94 kDa glucose-regulated protein (GRP94) (**B**); phosphorylated inhibitor of nuclear factor kappa-B kinase subunits alpha (IKKA) and beta (IKKB) (**C**), and nuclear factor kappa-B inhibitors alpha (IKBA) and beta (IBB) (**D**) were measured in muscles of untreated rats incubated with advanced glycated- (left muscle; AGE; black bars) or control- (right muscle; C; white bars) bovine albumin for 2.5 hours. On the left side of panels B, C and D, representative autoradiograms and Ponceau S staining of respective lanes, used as protein loading control, are shown. Data are mean ± SEM of 6 (p-IKKA, p-IKKB and IKKA); 7 (*Ager*, *Ddost* and IKKB); or 8 (GRP78 and GRP94) animals. Means were compared by paired two-tailed *t* test. *P < 0.05, **P < 0.01 and ***P < 0.001vs control-albumin.

In order to specify the role of AGE-albumin, the expression of AGE receptors was investigated. The results show that *Ager* mRNA expression increased in muscles incubated (2.5 hours) with AGE-albumin (Fig. 5A).

Two-and-a-half-hour incubation of muscles with AGE-albumin increased the expression of chaperones GRP78 and GRP94 proteins (Fig. 5B), revealing the activation of UPR. Besides, the results show that phosphorylation of IKKA and IKKB (Fig. 5C) increased, whereas the cellular content of IKBA and IKBB decreased (Fig. 5D), revealing the rapid activation of inflammatory pathway.

### *In vitro* treatment of rat skeletal muscle with AGE-albumin increased nuclear protein binding into NFKB-binding site of *Slc2a4* gene promoter

**Figure 6:**
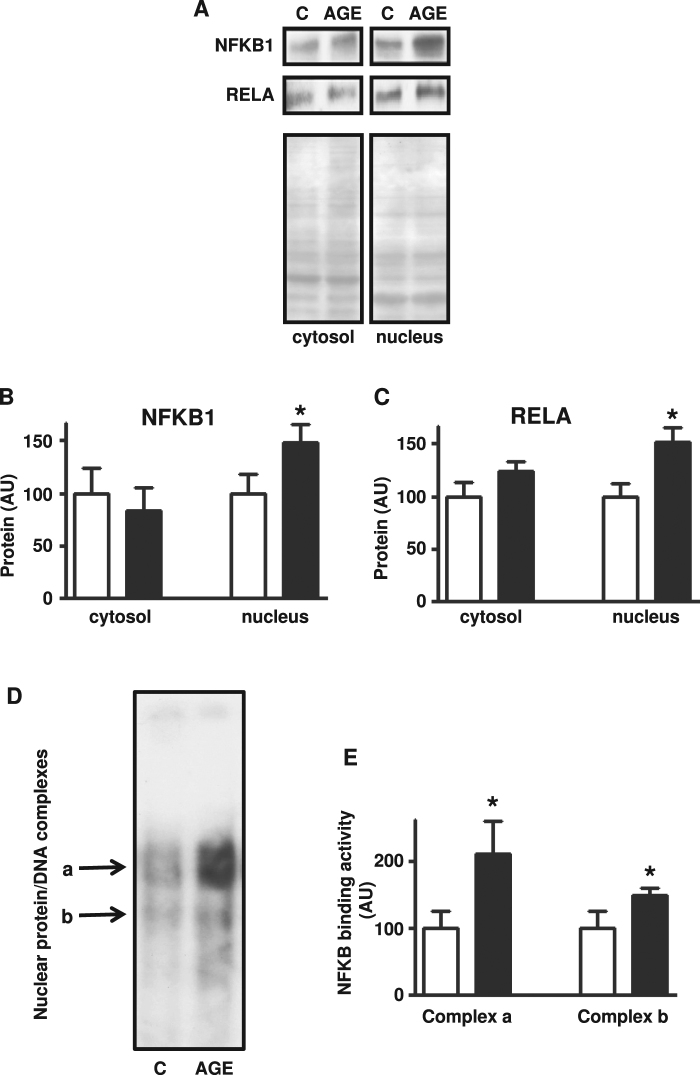
*In vitro* treatment of rat skeletal muscle with advanced glycated albumin increases nuclear content of NFKB1 and RELA proteins, and the nuclear proteins binding into *Slc2a4* promoter NFKB-binding site. Nuclear factor NF-kappa-B p50 protein (NFKB1) (**A**,**B**) and nuclear factor NF-kappa-B p65 protein (RELA) (**A**,**C**) proteins were measured in cytosolic and nuclear subcellular fractions. Nuclear protein binding into NFKB-binding site of *Slc2a4* gene promoter was analyzed by electrophoretic mobility shift assay and revealed two protein/DNA complexes *a* and *b* (**D**), which were separately quantified (**E**). Muscles were incubated for 2.5 hours with advanced glycated- (AGE, black bars) or control- (C, white bars) bovine albumin. In (**A**), representative autoradiograms and Ponceau S staining of respective lanes, used as protein loading control, are shown; and were cropped from different membranes. Data are mean ± SEM of 7 animals. Means were compared by paired two-tailed *t* test. *P < 0.05 vs control-albumin.

Firstly, we confirmed that the NFKB pathway activation culminates in increased nuclear content of both NFKB1 and RELA subunits of NFKB (Fig. 6A–C).

Once established that 2.5-hour incubation of muscle with AGE-albumin activated the NFKB inflammatory pathway, culminating with nuclear translocation of NFKB1 and RELA proteins, we investigated the nuclear protein binding into the NFKB-binding site of *Slc2a4* gene promoter region (Fig. 6D,E). As previously described^13^, two protein/DNA complexes were detected, and both complex “a” and complex “b” increased by 100% and 50%, respectively, in response to AGE-albumin.

## Discussion

AGEs are prevalent in DM and represent one of the basis for the development of long-term DM complications^17^. Albumin, the most abundant plasmatic protein, when modified by glycation displays an important role in cellular and tissue damage^18^. In the present study, we investigated whether glycated-albumin, regardless of a high glucose milieu, could modulate GLUT4 expression in skeletal muscle, which represents the latest downstream step for insulin-induced glucose disposal. In healthy subjects, skeletal muscle accounts for up to ~80% of glucose disposal under insulin-stimulated conditions, playing a fundamental role in glycemic homeostasis^19^. Regarding that, reduced GLUT4 expression in skeletal muscle was extensively reported in experimental models of type 2 DM (T2DM) in mice^20–24^. In human T2DM, although some pioneering studies have failed to detect reduced *SLC2A4*/GLUT4 expression in skeletal muscle^25–27^, this was firstly reported by Dohm and colleagues^28^, and further definitely confirmed by studies employing more sensitive analyses of GLUT4 quantification^8,29,30^. Furthermore, studies that have been investigated epigenetic regulation of *Slc2a4* gene in muscles from T2DM patients have now given attention for gene repression in this condition^30,31^.

According to the hypothesis that AGEs contribute to glycemic control impairment, reduced insulin-induced glucose uptake by skeletal muscle of animals fed a high AGE content diet was already reported^32,33^. Besides, by intraperitoneal injection of AGE-albumin, we have recently reported that AGEs impair whole-body insulin sensitivity^16^, which now can be ascribed to a reduction in skeletal muscle *Slc2a4*/GLUT4 expression.

In order to clarify the mechanisms potentially involved in this GLUT4 regulation, we firstly measured the expression of AGEs receptors *Ager* and *Ddost*. Treatment with AGE-albumin reduced the expression of *Ddost*, a regulation already described in mice subjected to chronic ingestion of oral AGEs^32^. Cellular effects of AGEs include a convergent activation of oxidative, ER and inflammatory stress^12^. Accordingly, AGE-albumin treated rats showed increased cellular GRP78 and nuclear NFKB1 content, respective markers of ER and inflammation stress, which can be responsible for impaired *Slc2a4*/GLUT4 expression.

In *in vitro* incubations, a direct effect of AGE-albumin in soleus muscle was demonstrated by the *Slc2a4* mRNA reduction after 2.5-hour AGE-albumin incubation, which reflected on decreased GLUT4 protein 2.5 hours later. Increased expression of *Slc2a*4 mRNA and GLUT4 protein has been observed as soon as 30 min to 120 min, respectively, after an enhancer stimulus^33–35^. However, decreasing effects have been described to occur later on; especially for GLUT4 protein, since the repressor effect depends not only on the transcriptional/translational inhibition, but also on the mRNA/protein half-life.

Decreased GLUT4 content was reported in muscle after 3-hour incubation with tumor necrosis factor alpha^36^, and in L6 muscle cells after 16-hour culture with linoleic or oleic fatty acids^37^. Here, GLUT4 protein reduction was detected 5 hours after muscle incubation with AGE-albumin, and that became more evident after 7.5 hours. These results can explain the AGE-induced reduction of glucose uptake described in L6 and in C2C12 cells after 8-hour AGEs treatment^3,38^.

Although the powerful effect of GLUT4 reduced expression in the AGE-induced impairment in muscle glucose disposal, we cannot discard the participation of impaired GLUT4 storage vesicles translocation to the plasma membrane. Reduced activity of some steps of insulin signaling pathway has been reported to occur in response to AGEs overload both *in vivo*^32,33^ and *in vitro*^36^. Considering that, we measured the 2-deoxy-D-glucose (2DG) uptake in muscles incubated *in vitro* with control- and AGE-albumin for 2.5 hours, a time point in which the total cellular GLUT4 content was not altered. No differences were observed in both basal and insulin-stimulated conditions, although the expected positive effect of insulin was clearly observed. This result indicates that, at least for 2.5 hours, the insulin-mediated traffic of GLUT4 storage vesicles was unaffected by AGEs, reinforcing the important role of *Slc2a4*/GLUT4 repressed expression.

*In vitro* incubation of soleus muscle confirmed that AGE-albumin can directly and rapidly activate ER and inflammatory stress, an effect that is probably related to the increased expression of the AGE receptor gene *Ager*. Muscle incubation with AGE-albumin for 2.5 hours increased GRP78 and GRP94 chaperones, evincing the initial activation of the UPR. Increased protein content of ER chaperone GRP78 has been observed in several tissues of 4-week high-AGEs fed mice^39^, but there is no report of this regulation in skeletal muscle. Curiously, the *in vivo* treatment with AGE-albumin did not alter GRP94; however, in lead- and polycystic ovary syndrome-induced UPR, the GRP78 increase was reported to be more significant than the GRP94 increase^40,41^.

As a direct effect and/or as an UPR-related effect, AGEs can induce inflammatory stress. *In vitro*, AGE-albumin induced a clear activation of the canonical NFKB pathway in muscle; an effect that culminated with increased nuclear content of NFKB1 and RELA proteins. Besides, electrophoretic mobility assay revealed an increased nuclear protein binding activity into a *Slc2a4* promoter NFKB-binding site. NFKB-mediated repression of *Slc24* expression was proposed to be an inflammatory effect of tumor necrosis factor alpha in adipocytes several years ago^42^. Only recently the NFKB repressor effect on *Slc2a4* transcription was finally confirmed^13^, in both adipose and muscle tissues. Furthermore, the repressor effect involves both NFKB1 (p50) and RELA (p65) proteins acting as a heterodimer^13^. Thus, the present data clearly show that AGEs, *in vitro*, repress *Slc2a4*/GLUT4 expression by a NFKB-mediated pathway. Although NFKB is a powerful repressor of *Slc2a4* transcription^13^, proposed to mediate AGEs effect in the present study, we cannot discard the possibility that AGEs also reduce the transcriptional activity of some *Slc2a4* enhancer.

The present data reveal that chronically administered AGE-albumin, regardless of a hyperglycemic condition, is able to impair glycemic homeostasis, by activating ER and inflammatory stress in skeletal muscle, which culminates with repression of *Slc2a4*/GLUT4 expression. This effect was also observed *in vitro*, in muscles from normal rats incubated with AGE-albumin for a few hours, in which the ER and inflammatory stress lead to increased NFKB1 and RELA binding activity into the *Slc2a4* promoter, thus explaining its gene transcription repression. These data reveal that AGEs may worsen glycemic control in diabetic subjects and impair glycemic homeostasis in non-diabetic states of increased AGEs concentration; and that involves an ER- and inflammatory-mediated repression of *Slc2a4*/GLUT4 expression.

Considering the high amount of AGEs in processed foods and high-fat diets, our results shed light on an important role of AGEs as inducers of insulin resistance, a key mechanism in the pathogenesis of T2DM. In this regard, strategies for AGEs intermediates detoxification and /or blocking AGEs signaling may be useful to prevent derangements in glucose homeostasis.

## Material and Methods

### Advanced glycation of albumin

The advanced glycation of rat (A6414; Sigma-Aldrich, Saint Louis, Missouri, USA) and bovine (A6003; Sigma-Aldrich, Saint Louis, Missouri, USA) albumin was performed *in vitro* by incubating albumin with freshly prepared 10 mM glycolaldehyde (Sigma Chemical Co., St. Louis, MO, USA) solution in phosphate buffer (PBS) at 37 °C, in a shaking water bath under N_2_ atmosfere, in the dark. Control albumin was incubated with PBS alone. Samples were extensively dialyzed against PBS and kept frozen at −80 °C until experiments. The amount of endotoxins was <50 pg endotoxin/mL as determined by the chromogenic Limulus amebocyte assay (Falmouth, MA, USA) (data not shown). Carboxymethyllysine determined by ELISA was 12.6 times greater in rat AGE-albumin as compared to C-albumin. In addition, carboxymethyllysine and pyrraline amounts (mmol/mmol of lysine) were determined by liquid chromatography-mass spectrometry being highly superior in glycated samples as compared to C, as previously described^43^.

### Animals

The *in vivo* effect of AGEs was investigated in four-week old male Wistar rats obtained from de Central Animal Facility of the University of São Paulo Medical School, and housed in controlled environment (12-h light/dark cycle), with chow diet and water *ad libitum*. Animals were randomized into two groups receiving daily intraperitoneal (i.p.) injections of 20 mg/kg/day of rat control- (C) or AGE-albumin^44^ for 12 weeks. At the end of week 12, animals were anesthetized via i.p. injection with sodium thiopental (60 mg/kg, Cristália, São Paulo, Brazil), and subjected to an insulin tolerance test or to blood (inferior vena cava) and soleus muscle (left and right) collection. Blood was processed for glucose and insulin concentration measurement^45^. The muscles were immediately frozen and stored at −80 °C for further analyses. This experimental protocol was approved by the Institutional Care and Research Advisory Committee (CAPPesq HC-FMUSP #002/14).

The *in vitro* effect of AGEs was investigated in soleus muscle harvested from untreated healthy control 65- to 75-day-old male Wistar rats (180 to 200 g body weight), obtained from the Animal Center of the Institute of Biomedical Sciences, University of São Paulo. Animals were housed under controlled conditions as described above. After i.p. anesthesia with thiopental sodium (60 mg/kg, Cristália, São Paulo, Brazil), left and right soleus muscles were harvested for the *in vitro* study. The experimental protocol was approved by the Ethical Committee for Animal Research of the Institute of Biomedical Sciences of the University of São Paulo (protocol #124/134/2).

All procedures performed on animals were in accordance with the relevant guidelines and regulations.

### Insulin tolerance test (ITT)

ITT was performed as previously described^45^. Tail blood samples were collected at 0, 4, 8, 12, 16 and 20 min after intravenous (penis vein) injection of regular insulin (0.75 U/kg, Humulin^®^ R, Eli Lilly and Company, Indianapolis, IN, USA). Blood glucose values from 4 to 20 min were transformed to Napierian logarithm and subjected to a linear regression. The slope of the regression was multiplied by −100, to express the result as %/min. The tests were performed from 9:00 to 11:00 hours, in 4-hour food deprived rats.

### *In vitro* muscle incubation

Both right and left soleus muscles were gently dissected to preserve the integrity of tendons. One tendon was fixed into a horizontal metallic support, whereas the other was connected to an isometric transducer by a pulley (TBM-4F, World Precision Instruments INC., Sarasota, FL, USA). Muscle length was adjusted to produce maximal twitch tension (~3 g). The muscles were immersed in 100 mL Krebs-Heinseleit buffer, pH 7.4, containing 8 mM D-glucose and 1 mg/mL of control- (right muscle) or AGE- (left muscle) bovine albumin. Muscles were incubated at 37 °C, continuously oxygenated with 95% O_2_: 5% CO_2_, for 2.5 hours, with buffer replacement every 1.25 hours. For GLUT4 protein analysis, incubation time was extended to 5 and to 7.5 hours, with buffer replacement every 2.5 hours. At the end of the incubation periods, muscles were immediately frozen and stored at −80 °C for further analyses.

### Muscle glucose uptake *in vitro*

*In vitro* muscle glucose uptake was evaluated using deoxy-D-glucose-2-[1,2-3 H(N)] (2DG), as previously described^24^. Soleus muscle strips were previously incubated for 1.25 hours with control- or AGE-albumin, as described above. After that, the strips were removed to flasks containing the same buffers (control- or AGE-albumin), added by 0.2 mCi/mL 2DG (deoxy-D-glucose-2-[1,2-3 H(N)], PerkinElmer, Boston, MA, USA), and with or without 400 mM insulin (Humulin® R, Eli Lilly, Indianapolis, IN, USA), and thus incubated for more 1.5 hours. This protocol totalizes 2.5 hours of exposition to the different albumins. Results were expressed as μmol/g tissue.

### Quantitative PCR analysis

*Slc2a4*, *Ddost* and *Ager* mRNAs, which codify GLUT4, DDOST and AGER proteins; respectively, were evaluated by reversed transcribed quantitative PCR (RT-qPCR). Total RNA was extracted by Trizol reagent (Invitrogen Life Technologies, Carlsbad, CA, USA) from muscles obtained after the *in vivo* and *in vitro* treatments. cDNA was obtained by reverse transcription, and then amplified using the StepOnePlus System (Life Technologies), and the following TaqMan Gene Expression Assays (Life Technologies): *Ddost* (Rn01518759_m1), *Ager* (Rn01525753_g1) and *B2m* (Rn00560865_m1). For rat *Slc2a4*, the non-inventoried primers 5′-GGCTGTGCCATCTTGATGAC-3′ (fw) and 5′-CACGATGGACACATAACTCATGGAT-3′ (rv); and the probe 5′-FAM-AACCCGCTCCAGCAGC-MGB3′ (Taqman, Life Technologies) were used (Poletto *et al*., 2015). Data were normalized by the expression of the housekeeping gene *B2m*. Relative levels of mRNA expression were calculated using the comparative cycle threshold (Ct) (2^−ΔΔCt^) method.

### Western blotting analyses

Total cellular membrane protein fraction for GLUT4 quantification was performed as previously described^35,37^. IKKA, IKKB, IKBA, IKBB, GRP78 and GRP94 were analyzed in a total cellular protein fraction: muscle samples were homogenized in buffer^20^, centrifuged at 12,000 g (at 4 °C, for 20 min), and the supernatants were subjected to electrophoresis. For NFKB1 and RELA protein analyses, cytosolic and nuclear protein fractions were obtained as previously described^45^. Protein concentration in the samples was determined by the Bradford method. Equal amounts of total protein (according to the target protein) were subjected to sodium dodecyl sulfated polyacrylamide gel electrophoresis, using 12% T and 2.7% C gel for GLUT4, NFKB1, RELA, IKBA and IKBB; and 8% T and 2,7% C gel for p-IKKA and p-IKKB. Proteins were then transferred to nitrocellulose membrane, and incubated with primary antibody against GLUT4 (rabbit anti-GLUT4, #07-1404, Millipore) phosphorylated-IKKA and -IKKB (rabbit anti-pIKKα/β Ser^180^/Ser^181^, sc-23470-R, Santa Cruz Biotechnology), IKBA (rabbit anti-IKBα, sc-371, Santa Cruz Biotechnology), IKBB (rabbit anti-IKBβ, sc-945, Santa Cruz Biotechnology), NFKB1 (goat anti-p50, sc-1190, Santa Cruz Biotechnology), RELA (rabbit anti-p65, ab-7970, Abcam), and GRP78/94 (mouse anti-KDEL, ADI-SPA-827-F, Enzo Lifesciences). The membrane was then incubated with horseradish peroxidase-linked secondary antibody, and signal was detected by chemiluminescence. Blots were quantified by optical densitometry (ImageScanner III, GE Healthcare, Uppsala, Sweden). Protein-loaded normalization was undertaken by analyzing the Ponceau-stained membrane^46^, and results were further normalized considering mean of control values as 100.

### Electrophoretic mobility shift assay (EMSA)

Nuclear proteins for EMSA were extracted from muscles incubated *in vitro*, and EMSA was performed as previously described^13^. The oligonucleotide used as probe corresponds to the -134/-113 sequence of the mouse *Slc2a4* gene, which was previously confirmed to bind NFKB1 and RELA using nuclear proteins from rat L6 muscle cells^13^. EMSA performed with this probe revealed two protein/DNA complexes (A and B) in rat muscle cells, and competition assays confirmed the presence of both NFKB1 and RELA in these complexes^13^.

### Statistical Analyses

Comparison of results from rats treated or not with rat AGE-albumin *in vivo* was performed by unpaired two-tailed *t* test, after confirmation that the variances were not significantly different. Comparison of results from muscles incubated (left) or not (right) with bovine AGE-albumin was performed by paired two-tailed *t* test. Glucose uptake was analyzed by one-way ANOVA. Differences were considered significant when *P* < 0.05, and the number of samples is informed in the legends.

### Data availability

There is no restriction on the availability of materials and data.
